# Using the Belinfante momentum to retrieve the polarization state of light inside waveguides

**DOI:** 10.1038/s41598-019-51028-9

**Published:** 2019-10-16

**Authors:** Vincent Ginis, Lulu Liu, Alan She, Federico Capasso

**Affiliations:** 1 Harvard John A. Paulson School of Engineering and Applied Sciences, Harvard University, 29 Oxford Street, Cambridge, MA 02138 USA; 2 Data Lab/Applied Physics, Vrije Universiteit Brussel, Pleinlaan 2, 1050 Brussel, Belgium

**Keywords:** Integrated optics, Nanophotonics and plasmonics, Micro-optics

## Abstract

Current day high speed optical communication systems employ photonic circuits using platforms such as silicon photonics. In these systems, the polarization state of light drifts due to effects such as polarization mode dispersion and nonlinear phenomena generated by photonic circuit building blocks. As the complexity, the number, and the variety of these building blocks grows, the demand increases for an *in-situ* polarization determination strategy. Here, we show that the transfer of the Belinfante momentum to particles in the evanescent field of waveguides depends in a non-trivial way on the polarization state of light within that waveguide. Surprisingly, we find that the maxima and minima of the lateral force are not produced with circularly polarized light, corresponding to the north and south poles of the Poincaré sphere. Instead, the maxima are shifted along the great circle of the sphere due to the phase differences between the scattered TE and TM components of light. This effect allows for an unambiguous reconstruction of the local polarization state of light inside a waveguide. Importantly, this technique depends on interaction with only the evanescent tails of the fields, allowing for a minimally invasive method to probe the polarization within a photonic chip.

## Introduction

The study of optical forces on particles sized roughly the wavelength of light began, essentially, with the work of Arthur Ashkin and the publication of his 1986 paper on the first optical trap^1^. Still a relatively young field of study^2^, the manipulation of matter using optical forces has thus far found relevance in optical cooling^3^, in the handling of biomolecules^4,5^, in high-precision force measurements^6^, and as a method of actuation^7–15^, sorting^16,17^, and self-assembly^18^ of particles and devices, among others.

**Figure 1:**
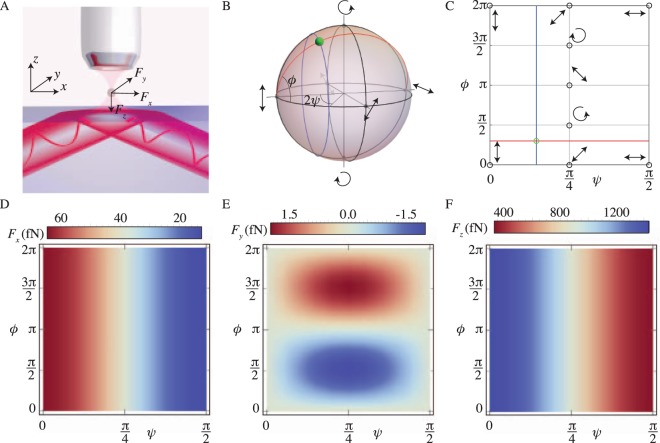
The different forces acting on a dielectric particle in the evanescent field above a surface. (**A**) Schematic description of our setup: the incident light (red beam) that generates the evanescent wave can have any polarization state represented by the green dot on the Poincaré sphere in (**B**). (**B**,**C**) together illustrate the mapping, Eq. (1), from the Poincaré sphere onto a rectangular grid. (**D**–**F**) show the forces that act on a 100 nm radius silicon particle on top of a silicon-water interface as a function of the incident polarization state. Simulations details of the incident Gaussian beam: power *P*_0_ = 100 *mW*, beam width *w*_0_ = 10 *μm*, incident angle *θ*_*in*_ = 45°, and free-space wavelength *λ*_0_ = 1.550 *μm*.

The optical forces acting on a microparticle in an evanescent field are visualized in Fig. 1A. For a Rayleigh particle, the force has two components: a gradient force (*F*_*z*_) that pulls the particle towards the region of highest field intensity, proportional to the gradient of the field, and a scattering force (*F*_*x*_) that pushes the particle along the propagation direction of the evanescent wave, proportional to the linear momentum of the wave^19^.

However, as the particle size increases, the interaction with the evanescent wave becomes more complex^20–24^. In the Mie regime, the scattering force is no longer entirely directed along the propagation direction of the evanescent wave. It obtains not only a component in the gradient *z* direction but, surprisingly, also a component in the out-of-plane *y* direction, whose sign depends on the helicity of the incident light^25–29^. This force is a consequence of the non-zero curl of the spatial profile of the spin angular momentum^30^ in an evanescent wave and is thus directly related to the Belinfante momentum^31^. Scientists have shown that these “lateral forces” can be found in a variety of configurations, using different types of particles and incident field distributions^32–40^.

In this letter, we investigate the lateral force acting on microparticles in the vicinity of an evanescent field. More specifically, we derive the general dependence of the forces as a function of the polarization state of the evanescent field and the optical properties of the interacting particles. We uncover a specific resonant phenomenon, where the size of the particle determines not only the amplitude of the forces, but also the polarization state, located on the Poincaré sphere, for which the maximal lateral force can be obtained. Finally, we show that this phenomenon can be used to measure the polarization state of light inside waveguides. Polarization is a slightly ambiguous concept for electromagnetic modes inside waveguides. In this context, it should be understood that polarization denotes the modal decomposition onto the waveguide TE and TM modes, which differs from the strict traditional interpretation of polarization in free space.

**Figure 2:**
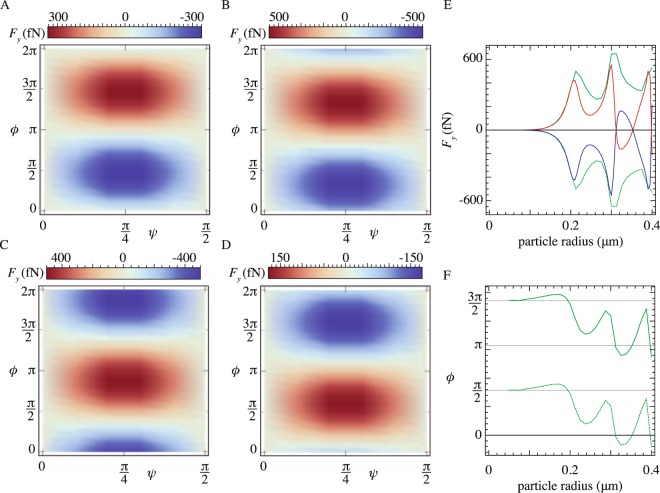
The dependence of the lateral force on the particle’s resonance dynamics. The lateral force acting on a silicon particle as a function of the polarization state of the incident beam for several radii: (**A**) *a* = 200 nm, (**B**) *a* = 250 nm, (**C**) *a* = 300 nm, (**D**) *a* = 350 nm. (**E**) The maximum and the minimum of the lateral force as a function of the particle radius (green lines), along with the force found at LCP (blue line) and RCP (red line). (**F**) The location on the Poincaré sphere–relative phase difference *ϕ*–where the maximum/minimum occurs. Simulations details identical to Fig. 1.

**Figure 3:**
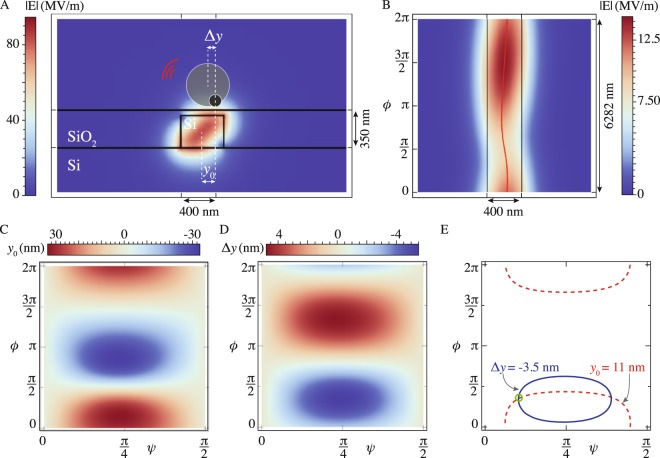
Using the lateral force for local, minimally invasive waveguide polarization sensing. (**A**) The cross section of a silica-embedded, rectangular silicon waveguide, showing the norm of the electric field. The measurement involves the comparison of the equilibrium locations of a small particle (*y*_0_) and a larger Mie particle (*y*_0_ + Δ*y*). *y*_0_ is defined as the shift of the 50 nm silicon particle away from the center of the waveguide. Δ*y* is defined as the displacement of the 300 nm particle relative to the 50 nm particle. (**B**) Topview of a waveguide, showing the oscillating behaviour of the field intensity along the propagation direction. The effective index of the TE and the TM mode equals 2.42 and 2.17, respectively. (**C**,**D**) *y*_0_ for a 50 nm silicon particle and Δ*y* for a 300 nm silicon particle as a function of the Poincaré plane. (**E**) The contours corresponding with a specific measurement of *y*_0_ and Δ*y*.

We start by considering a single interface between dielectric media. This analysis is shown in Figs 1 and 2. Subsequently, we add additional interfaces along the z- and the y-axis and simulate the force that is generated above a realistic waveguide. This is shown in Fig. 3.

To derive a general picture of the polarization-dependence of this lateral force, we expand the incident polarization in terms of the fundamental eigenmodes of the system (TE and TM polarized waves):

1 $${\overrightarrow{E}}_{{\rm{inc}}}=\,\cos (\psi ){\overrightarrow{E}}_{{\rm{TM}}}+\,\sin (\psi ){e}^{i\varphi }{\overrightarrow{E}}_{{\rm{TE}}}\mathrm{.}$$

Here, ${\overrightarrow{E}}_{{\rm{inc}}}$ refers to the incident field hitting the interface in the lower medium. ${\overrightarrow{E}}_{{\rm{TE}}}$ is oriented along the *y*-axis, while ${\overrightarrow{E}}_{{\rm{TM}}}$ contains both *x*- and *z*-components. The full expression of the evanescent field is given in the Supplemental Materials^19^.

Doing so, we introduce a convenient mapping of the Poincaré sphere on a two-dimensional plane, as visualized in Fig. 1B,C. Indeed, we can associate each state on the Poincaré sphere (green dot) with the two angles *ψ* and *ϕ*, as defined in Eq. (1). Note that, in this projection, light propagating through a wave-plate simply moves along one of the lines of fixed *ψ*. The same is true for an optical mode propagating in a birefringent waveguide. We evaluate the scattered fields of the dielectric sphere using Mie theory and calculate the forces directly from the Mie scattering coefficients, extending the formalism derived in ref.^22^ to permit arbitrary incident polarizations of light. The details of these calculations are given in the Supplemental Materials^19^. Fig. 1D–F shows the results of this calculation for the optical forces acting on a 100 nm radius silicon nanoparticle in an evanescent field of arbitrary polarization. Here, we are assuming the particle is 50 nm away from the interface. It is immediately clear that the forces in the *x* and the *z* directions do not depend on the relative phase difference *ϕ* between the TE and TM components of the polarization. There is, however, a nontrivial dependence of these forces as a function of the relative angle of the TE and TM component *ψ*. Importantly, the lateral force (*F*_*y*_) is the only component that is sensitive to the relative phase, *ϕ*, and, in this case, it reaches its maximum and minimum for purely left- and right-circularly polarized light (*ψ* = *π*/4, *ϕ* = ±*π*/2), in agreement with the theoretical prediction that the force is proportional to the helicity of the incident light.

However, this picture is not generally true. We demonstrate in Fig. 2 that the maximum and minimum of the lateral force, *F*_*y*_, are not necessarily located at the north and south poles of the Poincaré sphere, corresponding to purely right and left hand circularly polarized (RCP and LCP) light. Instead, the location of the maximum and minimum of the lateral force in the (*ψ*, *ϕ*) plane shifts in the *ϕ* direction as the particle size increases. Figure 2A–D demonstrates this shift for particles with radii 200 nm, 250 nm, 300 nm, and 350 nm, respectively. The other parameters in the model, such as wavelength, propagation constant, and refractive index, are fixed. Notice, for instance, the direction of the lateral force reverses for corresponding polarization states in Fig. 2A,D.

The physical origin of this shift is a phase difference between scattered TM and TE components of light, which manifests itself in the Mie regime, generating an extra contribution to the total helicity of the scattered light. In Fig. 2E, we highlight the difference between the maximal lateral force (green lines) and the lateral force that corresponds to the RCP and LCP polarization states (red/blue lines). For small particle sizes, this difference is negligible. But deeper into the Mie regime, the maximum lateral force oscillates as a function of particle size–a traditional Mie resonance signature. At the same time, the difference between the lateral force maximum/minimum and the force at LCP and RCP light increases. In Fig. 2F, we then plot the *ϕ* value on the Poincaré sphere, where the maximum and minimum in the lateral force occurs. The same resonant behavior is visible here: on resonance, the maxima and minima are shifted away from the south and north pole. To eliminate all other potential contributions to the helicity, we assumed, in these calculations, that the scattering phase at the interface is identical for both polarizations. A similar result is found when considering a traditional interface with asymmetric transmission coefficients. As shown in the Supplemental Material, differences in the transmission phases generate an extra shift in the *ϕ* direction on the Poincaré sphere^19^.

The specific phase sensitivity of the lateral force, and the manner in which it changes with probe size, is a unique feature that can be exploited for a minimally invasive measurement of the polarization state of light inside a waveguide. Indeed, in recent years, interest has grown in developing efficient techniques that allow for sensitive probing of the polarization state of light^41–49^.

A schematic of our technique is provided in Fig. 3A. Consider the equilibrium position of a small, Rayleigh particle above a waveguide. As a result of the birefringent nature of most rectangular waveguides, the modal field profile along a waveguide cross section is changing as the light propagates through the waveguide. In Fig. 3A, we plot a cross section of the electric field norm of a mode with *ψ* = *π*/4 and *ϕ* = 0, as obtained from full-wave numerical simulations (COMSOL)^19^. At the interface between the waveguide and its surroundings, the electric field is maximized at an offset *y*_0_ away from the center of the waveguide. This offset varies as a function of the relative amplitudes (*ψ*) of the TE and TM modes as well as the propagation distance inside the waveguide–or, equivalently, the phase difference (*ϕ*) between the two modes. This is visualized in Fig. 3B, where we plot the electric field strength along the top interface of the waveguide. The red line traces out the location of the electric field maximum. This line corresponds to the lateral equilibrium location of a small Rayleigh particle: although the spin-lateral force is present, it is negligible for a dipolar particle. In Fig. 3C, this equilibrium position, *y*_0_, of our small particle is visualized as a function of *ψ* and *ϕ*.

The function *y*_0_(*ψ*, *ϕ*) must be inverted to determine the polarization state within the waveguide. Most general displacements *y*_0_ map to a continuum of states describing a roughly elliptical contour line in Fig. 3C. To select the correct polarization state out of this continuum, one needs another piece of information. Because of its interaction with the Belinfante momentum, the displacement of a larger Mie particle will, in general, not be the same as the displacement of a small particle. Along with the gradient force which attracts the large particle towards the highest intensity position *y*_0_, there is a non-zero lateral force that pushes the particle away from the equilibrium position of the smaller particle, resulting in an additional displacement, Δ*y*. Although the details of this setup are different from the simple TIR-setup, analysed in Figs 1 and 2, we found that the same results remains valid in the case of a rectangular waveguide, where the linearly polarized vectors ${\overrightarrow{E}}_{TE}$ and ${\overrightarrow{E}}_{TM}$ need to be replaced by the fundamental even (TE-like) and odd (TM-like) modes of the waveguide, both characterized by spatially variant field profiles^19^. To find the equilibrium position of the larger particle (*y* = *y*_0_ + Δ*y*), one needs to balance the extra lateral force with the restoring force that pulls the particle towards the position of highest field intensity: Δ*y* = *F*_*y*_/*k*_*y*_. For a 300 nm radius silicon particle, Δ*y*(*ψ*, *ϕ*) is visualized in Fig. 3D. The two measurements, *y*_0_ (small particle) and Δ*y* (large particle), can be superimposed in the polarization plane (*ψ*, *ϕ*) as two non-overlapping contours. An example is shown in Fig. 3(E). The possible polarization is thus limited to one of the two intersection points of these contour lines. The correct intersection can be chosen using the asymmetry in scattering strength (see Fig. S3(H)) in combination with a calibration of the scattering intensity of light from the particle. As a result, the correct polarization state can be unambiguously extracted^19^. This measurement can be optimized by picking appropriate radii for the particles, generating a large phase mismatch between Fig. 3C,D.

In the simulations that are presented in this manuscript we have not included the effects of the trapping beam, which would be used to position the probe particles close to the surface. Although this beam would influence the dynamics of the probe particle, it should not influence its equilibrium location. The effects of the trapping beam can thus be eliminated using a calibration procedure that is typically used when making sensitive force measurements^29^. We also assumed that the effects of multiple reflections between the particle and the surface of the waveguide can be neglected. These reflections can give second-order contributions to evanescent field optical forces^50^.

In conclusion, our study shows that the recently discovered lateral force, acting on a Mie particle in an evanescent field, depends in a non-trivial way on the resonance dynamics of those particles. Interestingly, this behaviour can be used to retrieve the polarization state of light inside waveguides. The lateral force, acting on a particle in the evanescent field of a waveguide is highly polarization dependent and the equilibrium location of two particles with accurately chosen sizes in the vicinity of a waveguide thus allows for the unambiguous reconstruction of every possible polarization state inside the waveguide. Interestingly, we can reconstruct both amplitude and phase by simply relying on local measurements. In a traditional scattering NSOM experiment, e.g., the full polarization (amplitude and phase) of the field is found using interferometry of the wave scattered of the tip and a reference signal. The working wavelength throughout the paper is 1550 nm. It is important to note that the presented method can be used in any wavelength regime, assuming the waveguide has guided modes for that wavelength. In our analysis the waveguide is surrounded by water because this is the most convenient medium for a proof-of-concept experiment. In addition to the celebrated examples of actuation, sorting, and spectroscopy, we thus demonstrate that lateral forces can be used as a tool to develop a local, minimally invasive polarimetry probe.

## Supplementary information

Supplemental Material to: Using the Belinfante momentum to retrieve the polarization state of light inside waveguides
