# Supplementary material for: Using the Belinfante momentum to retrieve the polarization state of light inside waveguides
Source: Sci Rep. 2019 Oct 16;9:14879. doi: 10.1038/s41598-019-51028-9 (PMC6796005; doi:10.1038/s41598-019-51028-9)
Supplement: Supplementary file 1 — Supplemental Material to: Using the Belinfante momentum to retrieve the polarization state of light inside waveguides [file 41598_2019_51028_MOESM1_ESM.pdf]

Supplemental Material to: Using the Belinfante momentum to retrieve the polarization state of light inside waveguides

Vincent Ginis[1,2,*], **Lulu Liu**[1], **Alan She**[1], **and Federico Capasso**[1,+]

[1]Harvard John A. Paulson School of Engineering and Applied Sciences, Harvard University, 29 Oxford Street, Cambridge, MA 02138
[2]Data Lab / Applied Physics, Vrije Universiteit Brussel, Pleinlaan 2, 1050 Brussel, Belgium
[*]ginis@seas.harvard.edu
[+]capasso@seas.harvard.edu

ABSTRACT

Current day high speed optical communication systems employ photonic circuits using platforms such as silicon photonics. In these systems, the polarization state of light drifts due to effects such as polarization mode dispersion and nonlinear phenomena generated by photonic circuit building blocks. As the complexity, the number, and the variety of these building blocks grows, the demand increases for an in-situ polarization determination strategy. Here, we show that the transfer of the Belinfante momentum to particles in the evanescent field of waveguides depends in a non-trivial way on the polarization state of light within that waveguide. Surprisingly, we find that the maxima and minima of the lateral force are not produced with circularly polarized light, corresponding to the north and south poles of the Poincaré sphere. Instead, the maxima are shifted along the great circle of the sphere due to the phase differences between the scattered TE and TM components of light. This effect allows for an unambiguous reconstruction of the local polarization state of light inside a waveguide. Importantly, this technique depends on interaction with only the evanescent tails of the fields, allowing for a minimally invasive method to probe the polarization within a photonic chip.

1 Optical forces on Rayleigh particles in an evanescent field

The force acting on a Rayleigh particle in an evanescent field can be calculated analytically. The time-avaraged Lorentz force acting on such a spherical, dipolar particle with radius a consists of three terms[1]:

$$\vec{F} = \frac{\Re[\alpha]}{4}\nabla\|\vec{E}_0|^2 + \frac{\Im[\alpha]}{2}\vec{k}|\vec{E}_0|^2 - \frac{\Im[\alpha]}{2}\Im[\vec{E}_0\nabla\vec{E}_0]. \tag{S1}$$

Here, E_0 is the amplitude of the electric field with wave vector k, and α is the polarizability of the particle. For small particles ($ka \ll 1$) this polarizability can be approximated by $\alpha = \alpha_0\left(1+2/3ik^3|\alpha_0|^2\right)$ with $\alpha_0 = a^3(\varepsilon_r-1)/(\varepsilon_r+2)$, where $\varepsilon_r = \varepsilon_p/\varepsilon_b$ is the relative permittivity, ε_p the permittivity of the particle, and ε_b the permittivity of the background medium.

The first term in Eq. (S1) corresponds to the gradient force, whereas the second and the third term are scattering forces. The second term corresponds to the radiation pressure whereas the last term is proportional to the spin density of the field. We consider an evanescent field generated by an internally reflected TM-polarized plane wave (see Fig. 1 in the main paper). In this case, the first term pulls the particle towards the surface, the second one pushes the particle along the surface in the direction of propagation, and the final term depends on the curl of the helicity of light but remains negligible in the dipole approximation. In the case of linearly polarized light, the final term disappears altogether. The ratio of the forces perpendicular to (z direction) and longitudinal along the surface (x direction) is thus given by:

$$\left|\frac{F_z}{F_x}\right| = \frac{1\Re[\alpha]\nabla|\vec{E}_0|^2}{2\Im[\alpha]k_x|\vec{E}_0|^2}, \tag{S2}$$

where $\vec{E}_0 = E_0\vec{1}_E\exp[\mathrm{i}k_x\mathrm{x}-\mathrm{qz}]$. $|F_z/F_x|$ is thus entirely determined by the ratio of the real and the imaginary parts of the particle's polarizability α and the evanescent field's wave vector:

$$\left|\frac{F_z}{F_x}\right| = \frac{q}{k_x}\frac{\Re[\alpha]}{\Im[\alpha]}. \tag{S3}$$

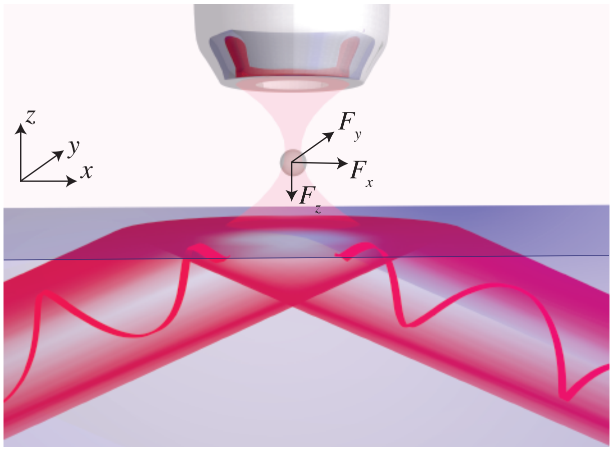

Figure S1. **A visualization of the forces acting on a particle in the evanescent field of a total interally reflected beam.** In the Rayleigh approximation, the gradient force pulls the particle in the z direction and the scattering force pushes the particle along the x direction. In the Mie regime, this picture become more complex and scattering forces appear in the three different directions. In a typical measurement of these forces, the particle is held at a fixed position using an optical trap (focused Gaussian beam from the objective)

The values of q and k_z are fixed by the angle of incidence. Using Draine's radiation correction for the polarizability of a small particle: $\alpha = \alpha_0 \left(1 - \frac{2}{3} ik^3 \alpha_0\right)^{-1} \approx \alpha_0 \left(1 + \frac{2}{3} ik^3 \alpha_0^*\right)$, one finds that, for lossless particles, the ratio of the real and the imaginary parts of the polarizability equals

$$\frac{\Re[\alpha]}{\Im[\alpha]} = \left(\frac{2}{3} k^3 \alpha_0\right)^{-1}. \tag{S4}$$

The ratio of the force in the z and the x direction grows to infinity for decreasing particles radius a, as the static polarizability α_0 is proportional to a^3.

2 Optical forces on Mie particles in an evanescent field

When the size of a dielectric particle is comparable to the wavelength of the incident light, the Rayleigh approximation is not valid anymore. In this case, one needs to solve the full electromagnetic problem, calculating the scattered fields imposing Maxwell's boundary equations at the surface of the particle. For homogeneous, isotropic, spherical particles, this problem was solved by Gustaaf Mie in 1908 and is now known as the generalized Lorenz-Mie theory[2].

The formalism starts by decomposing the incident fields—with subscript (i)—as a sum of spherical harmonics:

$$E_r^{(i)} = \frac{a^2 E_0}{r^2} \sum_{l=1}^{\infty} \sum_{m=-l}^{l} l(l+1) A_{l,m} \psi_l(n_b k_0 r) Y_{l,m}(\theta, \phi), \tag{S5}$$

$$H_r^{(i)} = \frac{a^2 H_0}{r^2} \sum_{l=1}^{\infty} \sum_{m=-l}^{l} l(l+1) B_{l,m} \psi_l(n_b k_0 r) Y_{l,m}(\theta, \phi). \tag{S6}$$

Here, a is the radius of the particle, n_b is the refractive index of the background, k_0 is the free space wave vector ($k_0 = \omega/c$), E_0 and H_0 are the amplitudes of the electric and magnetic fields, $Y_{\mathrm{l,m}}(\theta, \phi)$ are the spherical harmonics, and $\psi_l(r) = r j_l(r)$ are the Riccati-Bessel functions of the first kind. These functions are defined as $\psi_l(r) = r j_l(r)$, with $j_l(x)$ the spherical Bessel function of the first kind. It is sufficient to consider only the radial parts of the electromagnetic field to carry out this decomposition, as the other components are directly related to the radial ones through Maxwell's equations. Using the orthogonality of the spherical harmonics, the coefficients $A_{l,m}$ and $B_{l,m}$ can be retrieved by evaluation of the following integrals at the surface of the particle:

$$A_{l,m} = \frac{C}{E_0} \int_0^{2\pi} \int_0^{\pi} E_r^{(i)} Y_{l,m}^*(\theta, \phi) \sin\theta \, \mathrm{d}\theta \mathrm{d}\phi, \tag{S7}$$

$$B_{l,m} = \frac{C}{H_0} \int_0^{2\pi} \int_0^{\pi} H_r^{(i)} Y_{l,m}^*(\theta, \phi) \sin\theta \, \mathrm{d}\theta \mathrm{d}\phi, \tag{S8}$$

where $C = (l(l+1)\psi_l(n_b k_0 a))^{-1}$.

Next, one considers a similar decomposition of the scattered fields—with subscript (s). However, in order to impose the correct boundary conditions at infinity, one traditionally uses modified Riccati-Bessel functions $\xi(r) = rh_l^{(1)}(r)$, where $h_l^{(1)}(r)$ are the spherical Hankel functions of the first kind:

$$E_r^{(s)} = \frac{a^2 E_0}{r^2} \sum_{l=1}^{\infty} \sum_{m=-l}^{l} l(l+1) a_{l,m} \xi_l(n_b k_0 r) Y_{l,m}(\theta,\phi), \tag{S9}$$

$$H_r^{(s)} = \frac{a^2 H_0}{r^2} \sum_{l=1}^{\infty} \sum_{m=-l}^{l} l(l+1) b_{l,m} \xi_l(n_b k_0 r) Y_{l,m}(\theta,\phi). \tag{S10}$$

In general, one would have to write a similar decomposition for the fields inside the particle and subsequently impose the continuity of the tangential parts of the fields at the boundary of the particle. Following this procedure, Gustav Mie derived a closed form expression for the scattered fields as a function of the incident fields, or equivalently, the scattered coefficients $a_{l,m}$ and $b_{l,m}$ as a function of the incident coefficients $A_{l,m}$ and $B_{l,m}$:

$$a_{l,m} = \frac{n_b \psi_l(k_b a)\psi_l'(k_p a) - n_p \psi_l(k_p a)\psi_l'(k_b a)}{n_p \psi_l(k_p a)\xi_l'(k_b a) - n_b \xi_l(k_b a)\psi_l'(k_p a)} A_{l,m}, \tag{S11}$$

$$b_{l,m} = \frac{n_p \psi_l(k_b a)\psi_l'(k_p a) - n_b \psi_l(k_p a)\psi_l'(k_b a)}{n_b \psi_l(k_p a)\xi_l'(k_b a) - n_p \xi_l(k_b a)\psi_l'(k_p a)} B_{l,m}, \tag{S12}$$

where $k_b = n_b k_0$ and $k_p = n_p k_0$.

At this point, the full electromagnetic problem is solved and the time-avaraged force acting on the particle can, in principle, be calculated by integrating the flux of the Maxwell stress tensor over a surface that surrounds the particle:

$$< F_i > = < \oint T_{ij} n_j \mathrm{d}S >, \tag{S13}$$

where

$$T_{ij} = \varepsilon_0 \left(E_i E_j - \frac{1}{2}\delta_{ij} E^2 \right) + \frac{1}{\mu_0} \left(B_i B_j - \frac{1}{2}\delta_{ij} B^2 \right). \tag{S14}$$

Unfortunately, this procedure is rather cumbersome as the integral converges slowly for larger particles and is very sensitive to discretization error. In order to avoid this last integration step, Barton et al. derived a formula that allows for the calculation of the force using an algebraic combination of the Mie scattering coefficients[3]. Using this algorithm, we calculated the time-averaged force as

$$
\begin{aligned}
\frac{\langle F_x\rangle}{a^2\varepsilon_0E_0^2} =&\mathrm{Im}[\frac{-\alpha^2}{2}\sum_{l=1}^{\infty}\sum_{m=-l}^{l}\sqrt{\frac{(l-m+1)(l+m+1)}{(2l+1)(2l+3)}}l(l+2)\\
&\times(2n_b^2a_{l+1,m}a_{l,m}^*+n_b^2a_{l+1,m}A_{l,m}^*+n_b^2A_{l+1,m}a_{l,m}^*\\
&\quad+2b_{l+1,m}b_{l,m}^*+b_{l+1,m}B_{l,m}^*+B_{l+1,m}b_{l,m}^*\\
&\quad+n_bm(2a_{l,m}b_{l,m}^*+a_{l,m}B_{l,m}^*+A_{l,m}b_{l,m}^*))].
\end{aligned}
\tag{S15}
$$

$$
\begin{aligned}
\frac{\langle F_y\rangle}{a^2\varepsilon_0E_0^2} =&\mathrm{Im}[\frac{\mathrm{i}\alpha^2}{4}\sum_{l=1}^{\infty}\sum_{m=-l}^{l}\sqrt{\frac{(l+m+2)(l+m+1)}{(2l+1)(2l+3)}}l(l+2)\\
&\times(2n_b^2a_{l,m}a_{l+1,m+1}^*+n_b^2a_{l,m}A_{l+1,m+1}^*+n_b^2A_{l,m}a_{l+1,m+1}^*\\
&\quad+2b_{l,m}b_{l+1,m+1}^*+b_{l,m}B_{l+1,m+1}^*+B_{l,m}b_{l+1,m+1}^*)\\
&+\sqrt{\frac{(l-m+1)(l-m+2)}{(2l+1)(2l+3)}}l(l+2)\\
&\times(2n_b^2a_{l+1,m-1}a_{l,m}^*+n_b^2a_{l+1,m-1}A_{l,m}^*+n_b^2A_{l+1,m-1}a_{l,m}^*\\
&\quad+2b_{l+1,m-1}b_{l,m}^*+b_{l+1,m-1}B_{l,m}^*+B_{l+1,m-1}b_{l,m}^*)\\
&-\sqrt{(l+m+1)(l-m)}n_b\\
&\times(-2a_{l,m}b_{l,m+1}^*+2b_{l,m}a_{l,m+1}-a_{l,m}B_{l,+1}^*\\
&\quad+b_{l,m}A_{l,m+1}^*+B_{l,m}a_{l,m+1}^*-A_{l,m}b_{l,m+1}^*)],
\end{aligned}
\tag{S16}
$$

$$
\begin{aligned}
\frac{\langle F_z\rangle}{a^2\varepsilon_0E_0^2} =&\mathrm{Re}[\frac{\mathrm{i}\alpha^2}{4}\sum_{l=1}^{\infty}\sum_{m=-l}^{l}\sqrt{\frac{(l+m+2)(l+m+1)}{(2l+1)(2l+3)}}l(l+2)\\
&\times(2n_b^2a_{l,m}a_{l+1,m+1}^*+n_b^2a_{l,m}A_{l+1,m+1}^*+n_b^2A_{l,m}a_{l+1,m+1}^*\\
&\quad+2b_{l,m}b_{l+1,m+1}^*+b_{l,m}B_{l+1,m+1}^*+B_{l,m}b_{l+1,m+1}^*)\\
&+\sqrt{\frac{(l-m+1)(l-m+2)}{(2l+1)(2l+3)}}l(l+2)\\
&\times(2n_b^2a_{l+1,m-1}a_{l,m}^*+n_b^2a_{l+1,m-1}A_{l,m}^*+n_b^2A_{l+1,m-1}a_{l,m}^*\\
&\quad+2b_{l+1,m-1}b_{l,m}^*+b_{l+1,m-1}B_{l,m}^*+B_{l+1,m-1}b_{l,m}^*)\\
&-\sqrt{(l+m+1)(l-m)}n_b\\
&\times(-2a_{l,m}b_{l,m+1}^*+2b_{l,m}a_{l,m+1}-a_{l,m}B_{l,+1}^*\\
&\quad+b_{l,m}A_{l,m+1}^*+B_{l,m}a_{l,m+1}^*-A_{l,m}b_{l,m+1}^*)],
\end{aligned}
\tag{S17}
$$

The infinite series in the Mie scattering algorithm was truncated at a value l for which the relative magnitude of the different force contributions dropped below 10^{-7}.

3 The location of the maximum of the lateral force

It is interesting to take a closer look at the location on the Poincaré sphere where the maximal/minimal lateral force appears. In Fig. 2F of the main paper, we plot the angle ϕ of this polarization as a function of particle size. This angle uniquely defines the location because the polarization that generates the largest lateral force always lies on the great circle defined by $\psi=\pi/4$. Physically, this can be understood because of the fact that the lateral force is proportional to the product of the amplitudes of the TE- and the TM-polarizations. Recalling the definition of ψ and ϕ,

$$\vec{E}_{\mathrm{inc}}=\cos(\psi)\vec{E}_{\mathrm{TM}}+\sin(\psi)\exp(\mathrm{i}\phi)\vec{E}_{\mathrm{TE}}, \tag{S18}$$

we find that the force is proportional to $\sin(2\psi)$, which is maximal at $\psi=\pi/4$.

It is also intriguing to note that the different transmission amplitudes for TE- and TM-polarizations at the interface only affect the ϕ component of the polarization maximum/minimum. Indeed, the electric field above the interface can be written as:

$$\vec{E}_{\mathrm{interface}}=\cos(\psi)t_{\mathrm{TM}}\vec{E}_{\mathrm{TM}}+\sin(\psi)\exp(\mathrm{i}\phi)t_{\mathrm{TE}}\vec{E}_{\mathrm{TE}}, \tag{S19}$$

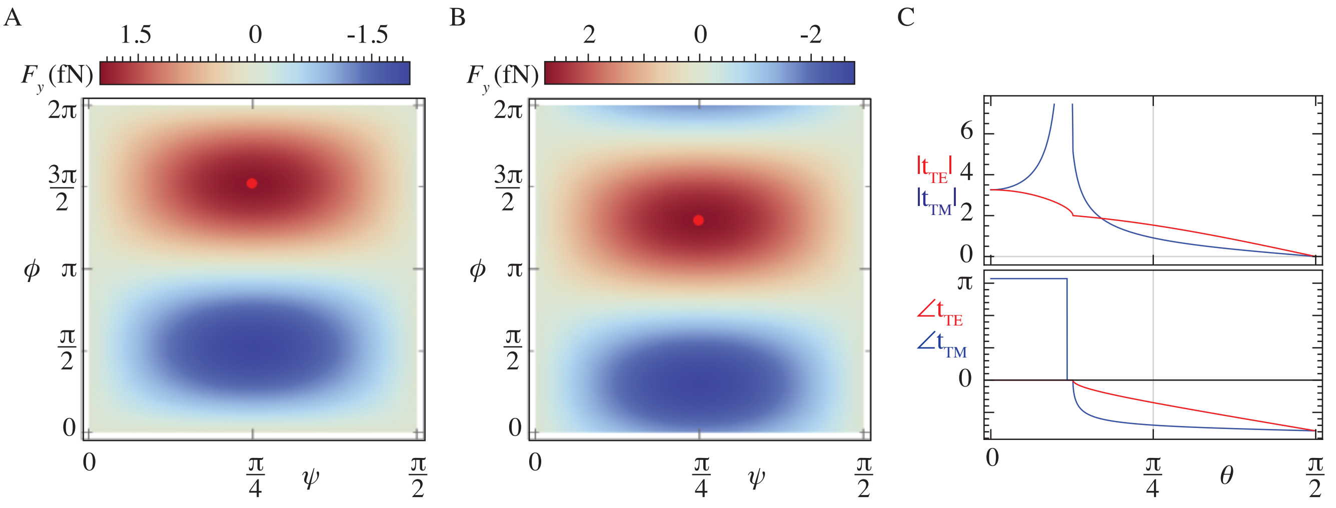

Figure S2. The effect of different transmission coefficients t_{TE} an t_{TM} at the interface on the location of the maximal lateral force. (A) The lateral force acting on a 100 nm radius silicon particle (λ = 1550 nm, $\theta = \pi/4$) assuming the transmission coefficients at the interface are identical. (B) Same setup as in (A), but in this case, the transmission coefficients are implemented conform the Fresnel equations. The location of the polarization state that corresponds to the maximum lateral force is shifted in the ϕ direction.This shift is perfectly in agreement with the difference between the arguments of the transmission coefficients at the angle of incidence $\theta = \phi/4$. (C) The magnitude and phase of the transmission coefficients versus angle of incidence θ.

where t_{TM} and t_{TE} are the transmission coefficients at the interface. It is interesting to note here that the amplitudes of the $\vec{E}_{TE}$ and the $\vec{E}_{TM}$ modes above the surface are determined by the parameter ψ, on the one hand, and the Fresnel transmission coefficients, on the other hand. However, the product of the TE and TM components is still maximized at $\psi = \pi/4$. The phase difference between the TE and TM transmission coefficients shifts the location of the maximum on the Poincaré sphere in the ϕ direction. This is visualized in Fig. S2, where we compare the lateral forces that act on a silicon particle ($a = 100nm$) between (A) a setup where the transmission coefficients are identical and (B) a setup where the transmission coefficients are given by the normal Fresnel values. The location of the maximal lateral force (red dot in both plots) is given by ($\pi = \pi/4$, $\phi = 1.52\pi$) in (A) and by ($\pi = \pi/4$, $\phi = 1.30\pi$) in (B). The shift of this location in the ϕ direction is in agreement wit the phase difference between t_{TE} and t_{TM} at $\theta = \pi/4$, where $\arg(t_{\mathrm{TE}}) - \arg(t_{\mathrm{TE}}) = 0.22\pi$.

4 The evanescent field in Cartesian coordinates

In order to calculate the force components acting on a dielectric particle in the Mie regime, it is convenient to write the evanescent field in Cartesian coordinates. We can rewrite Eq. (S19) by expressing $\vec{E}_{\mathrm{TE}}$ and $\vec{E}_{\mathrm{TE}}$ as a function of $\vec{1}_{\mathrm{x}}$, $\vec{1}_{\mathrm{y}}$, and $\vec{1}_{\mathrm{z}}$, using the expressions

$$\vec{E}_{\mathrm{TE}} = E_{\mathrm{TE}}\vec{1}_{\mathrm{TE}}, \tag{S20}$$

$$\vec{E}_{\mathrm{TM}} = E_{\mathrm{TM}}\vec{1}_{\mathrm{TM}}, \tag{S21}$$

$$\vec{1}_{\mathrm{TE}} = \vec{1}_{\mathrm{y}}, \tag{S22}$$

$$\vec{1}_{\mathrm{TM}} = \mathrm{i}\sqrt{\left(\frac{n_1}{n_2}\right)^2 \sin^2\theta_{in} - 1}\,\vec{1}_{\mathrm{x}} + \left(\frac{n_1}{n_2}\right)\sin\theta_{in}\vec{1}_{\mathrm{z}}. \tag{S23}$$

The full expression of the electric field above the interface is thus given by

$$\vec{E}_{\mathrm{interface}} = \cos(\psi)t_{\mathrm{TM}}E_{\mathrm{TM}}\left(\mathrm{i}\sqrt{\left(\frac{n_1}{n_2}\right)^2 \sin^2\theta_{in} - 1}\,\vec{1}_{\mathrm{x}} + \left(\frac{n_1}{n_2}\right)\sin\theta_{in}\vec{1}_{\mathrm{z}}\right) + \sin(\psi)\exp(\mathrm{i}\phi)t_{\mathrm{TE}}E_{\mathrm{TE}}\vec{1}_{\mathrm{y}}. \tag{S24}$$

5 Equilibrium position of particles above a waveguide

In this section, we discuss the equilibrium position of a particle above a (birefringent) waveguide in detail. The geometry of the waveguide, shown in Fig. 4 of the main paper, consists of a silicon core (400 nm wide, 300 nm high) and a silica cladding. The lateral equilibrium position (y) of a particle above the waveguide is essentially determined by two different forces: (1), a gradient force, attracting the particle to the position of largest intensity, and (2), the helicity dependent lateral force (caused by the Belinfante momentum).

Let us first consider the gradient force. This force essentially appears because of the "snaking" intensity profile of the field above the waveguide. This is a result of the different propagation constants of the different modes inside the waveguide, in combination with the fact that the field intensity above the waveguide shifts when you add the different modes with with different weights (ψ) and different relative phase (ϕ). To obtain this profile, we calculate the field amplitude at the interface of the waveguide using full wave numerical simulations (COMSOL Multiphysics). In Fig. S3(A-C), we show a few examples of these profiles for different values of ψ and ϕ. It is clear that the maximum of the field profile shifts as a function of the polarization state inside the waveguide. This equilibrium location (y_0) was extracted for each different polarization state of light inside the waveguide and is visualized in Fig. S3(G).

The second contribution is the helicity-dependent lateral force. In contrast to the gradient force, this force is only non negligible for larger particles, with radii in the Mie regime. This extra lateral force acting on 300 nm radius silicon particles was calculated using the Mie scattering algorithm, where the incident wave is now a superposition of the two evanescent waves of the different modes inside the waveguide. This result is shown in Fig. S4(A). It is interesting to note that there is an asymmetry in this density plot, displaying larger positive forces than negative forces. This can be understood when we look the intensity of the mode profiles above the waveguide as a function of the polarization state, as shown in Fig. S3(H). The asymmetry of the lateral force is thus entirely determined by the differences in intensity of the light above the waveguide. In order to find the overal displacemnt of the 300 nm radius particle, we still need to know how large the spring constant is of the gradient force attracting the particle towards the largest intensity. This spring constant is also a function of the polarization of light. Using the gradients the field cross sections (Fig S3(D-F)), we calculated this spring constant as shown in Fig. S3(I). Not surprisingy, the polarization regions with largest field amplitudes in Fig. S3(H) are also the polarization regions with the largest spring constants in Fig. S3(I). As a result, we notice that the extra displacement $\Delta y = F_{\text{lat}}/k_y$, shown in Fig. S4(B) is again more symmetric. Finally, in Fig. S4(C), we plot the overlap of the contour lines of y_0 and Δ_y, demonstrating that the points of intersection allow for the retrieval of the polarization state inside the waveguide. In general, the two roughly elliptical contour lines will have two intersections, from which the correct intersection can be chosen using the asymmetry of Fig. S3(H) in combination with a calibration of the scattering intensity of light from the particle.

We acknowledge the support of NSF GFRP grant number DGE1144152 and the Research Foundation Flanders grant number 12O9115N.

References

1. Albaladejo, S., Marqués, M. I., Laroche, M. & Sáenz, J. J. Scattering forces from the curl of the spin angular momentum of a light field. *Phys. review letters* **102**, 113602 (2009).

2. van de Hulst, H. C. *Light Scattering by Small Particles* (John Wiley Sons, Inc., NY, 1957).

3. Barton, J., Alexander, D. & Schaub, S. Theoretical determination of net radiation force and torque for a spherical particle illuminated by a focused laser beam. *J. Appl. Phys.* **66**, 4594–4602 (1989).

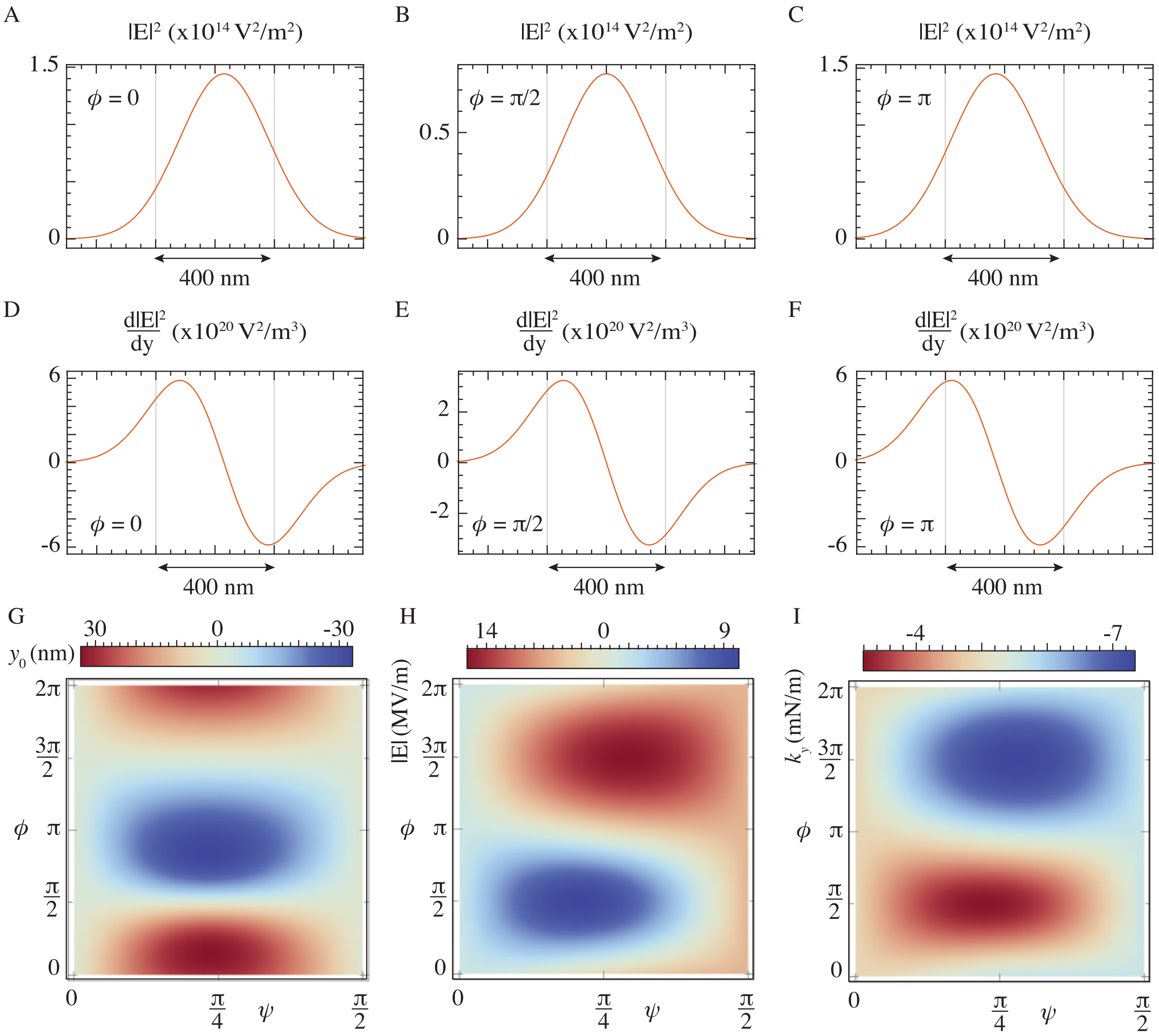

Figure S3. **The field profile above the surface of the waveguide determines the equilibrium position of small particles** (A)-(C) The intensity profile above a waveguide as a function of the polarization state inside the waveguide (A: $\psi = \pi/4$, $\phi = 0$; B: $\psi = \pi/4$, $\phi = \pi/2$; C: $\psi = \pi/4$, $\phi = \pi$) (D)-(F) The derivative of the field intensity (with same parameters as in (A)-(C)). (G) The equilibrium location of a small particle above the waveguide versus polarization state. H. The maximal amplitude of the field above the waveguide as a function of polarization state. (I) The spring constant of the potential attracting the particles towards the highest intensity as a function of the polarization state.

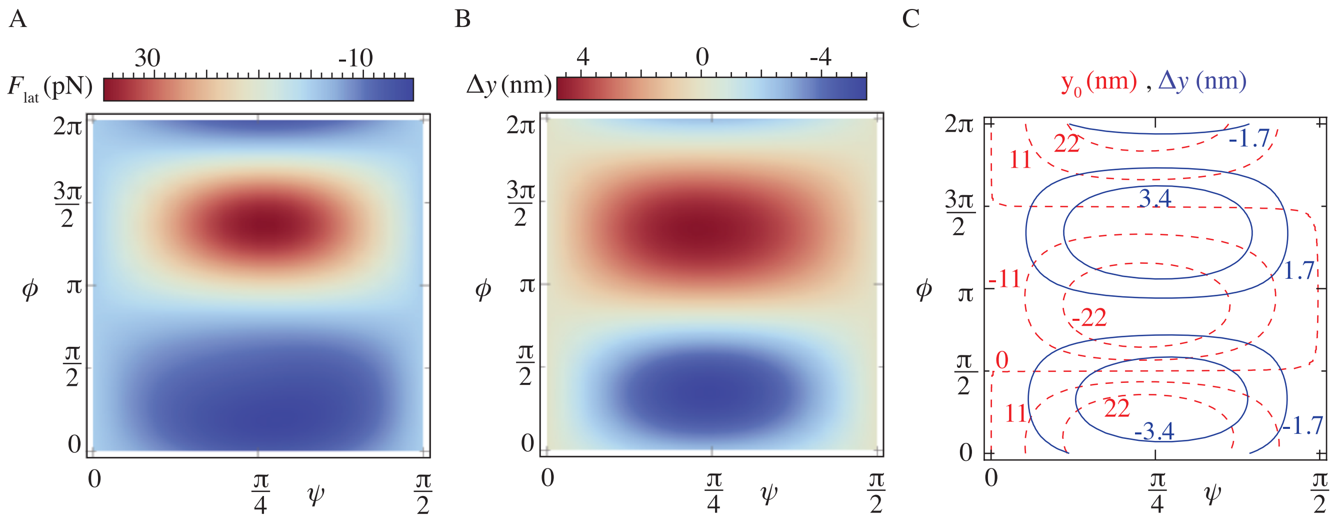

Figure S4. **Extracting the polarization state of light using the extra lateral displacement of a Mie particle on top of a waveguide.** (A) The helicity-dependent lateral force acting on a 300 nm radius silicon particle on top of a birefringent waveguide. (B) The displacement of the particle, found by balancing the lateral force with the spring force of the intensity potential pulling the particle towards the largest intensity: $\Delta y = F_{\mathrm{lat}}/k_y$. (C) The overlap of several contour lines of y_0 and Δy. Because of the relative displacement of these contour lines in the ϕ direction, we generally find only two intersections per set of two measurements. The correct intersection can be retrieved using a calibration of the scattering intensity.
